# Effects of the Chinese herbal formula San-Huang Gu-Ben Zhi-Ke treatment on stable chronic obstructive pulmonary disease: study protocol of a randomized, double-blind, placebo-controlled trial

**DOI:** 10.1186/s13063-019-3729-1

**Published:** 2019-11-27

**Authors:** Yaqi Zu, Demin Li, Xiang Lei, Hongchun Zhang

**Affiliations:** 1 Beijing University of Chinese Medicine, Chao Yang District, Beijing, 100029 People’s Republic of China; 2 Respiratory Department of Traditional Chinese Medicine, China-Japan Friendship Hospital, 2 Cherry Garden East Street, Chaoyang District, Beijing, 100029 China; 3 Beijing Qihuang Pharmaceutical Clinical Research Center, Feng Tai District, Beijing, China; 4 Department of Chinese Medicine Office, China-Japan Friendship Hospital, 2 Cherry Garden East Street, Chaoyang District, Beijing, 100029 China

**Keywords:** Chronic obstructive pulmonary disease, Traditional Chinese medicine, Chinese herbal formula San-Huang Gu-Ben Zhi-Ke, Clinical trials, Clinical protocols

## Abstract

**Background:**

Due to the large number of patients, high mortality rate as well as high social costs and economic burden, chronic obstructive pulmonary disease (COPD) has become one of the most important health problems around the world, which has attracted people’s attention. Currently, Chinese herbs have been widely used as alternative medicine (CAM) for COPD patients. The Chinese herbal formula San-Huang Gu-Ben Zhi-Ke (SHGBZK) has shown good clinical efficacy in COPD in preclinical studies. Animal experiments have shown that it has mucosal immune barrier function and can maintain airway wall integrity, reduce inflammatory cell infiltration, promote inflammatory damage repair, and relieve narrow airway conditions.

**Methods/design:**

This study is a randomized, double-blind, placebo-controlled trial. A total of 100 patients with stable COPD diagnosed with deficiency of lung qi and spleen qi will be recruited and randomly assigned to one of two treatment groups: SHGBZK treatment, *N* = 50; placebo treatment, N = 50. The two groups will receive basic treatment for COPD according to the 2017 GOLD Guidelines for Chronic Obstructive Pulmonary Disease. Patients will stick to the treatment they used to take as much as possible, and will be given present general treatment when acute exacerbation of COPD occurs during the study. Both groups will receive a 24-week intervention and patient status will be assessed at 24 weeks and then 28 weeks after treatment. After the 24-week treatment, patients will be followed up for another 28 weeks. Outcome measures, including the frequency and duration of acute exacerbation, lung function, traditional Chinese medicine symptom score, exercise capacity, and quality of life will be assessed.

**Discussion:**

It is hypothesized that SHGBZK will have beneficial effects in reducing the frequency and duration of acute exacerbations, improving the exercise capacity function of patients with stable COPD diagnosed with a deficiencies in lung qi and spleen qi. This study may establish a new treatment method for COPD patients, differentiating it from other drugs in clinical use used for similar clinical indications.

**Trial registration:**

Chinese Clinical Trial Registry, ChiCTR1800016349. Registered on 26 May 2018.

## Background

Chronic obstructive pulmonary disease (COPD) is characterized by persistent and limited airflow, which can be progressively aggravated. The repeated acute exacerbation of COPD (AECOPD) can lead to a variety of complications that lead to the disease worsening [1]. Reducing AECOPD is a major goal of COPD management and an important indicator for evaluating treatments. According to statistics [2], the global incidence of COPD is about 10% of the population. It is expected that COPD will have the third highest fatality rate in the world [3] and its economic burden will rank fifth in the world by 2020. In China, the prevalence of COPD in people over 40 years old is 8.2% [4], the number of deaths due to COPD exceeds one million each year, and the number of disabled people over five million. In addition to pulmonary symptoms, studies have shown that the most common complications of COPD patients are cardiovascular disease, diabetes, asthma, and anemia [5]. Most patients have one or two complications, which increase the social and economic burden. And most patients have mental anxiety symptoms [6], which seriously affect their working ability and quality of life [7]. As COPD can be prevented, effective preventive measures will help to delay the recurrence and progressive aggravation of the disease.

Current treatments for COPD include inhaled corticosteroids and bronchodilators [8]. Although effective at alleviating symptoms, these treatment methods do not alter disease progression. There is a pressing need to find better treatments [9] for improving clinical symptoms, lowering lung function decline, reducing mortality, and shortening hospitalization time. Traditional Chinese medicine (TCM) has a great advantage in reducing the risk for severe exacerbation, improving lung function, positively impacting quality of life, and improving exercise capacity in stabilized patients with COPD, which can supplement the deficiency of modern medical treatment [10].

SHGBZK Chinese medicine is a Chinese herbal formula developed by Professor Chao Enxiang, a great doctor of Chinese medicine with more than 50 years of clinical experience. Preliminary clinical data demonstrated that it has good clinical efficacy in COPD and no obvious side effects [11]. Animal experiments have shown that it has mucosal immune barrier function and can maintain airway wall integrity, reduce inflammatory cell infiltration, promote inflammatory damage repair, and relieve airway narrow conditions [12]. This study aims to evaluate the efficacy and safety of SHGBZK Chinese medicine for further research into developing a new, safe, and effective method for the treatment of stable COPD.

### Study objectives

This study will evaluate the safety and efficacy of the Chinese herbal formula SHGBZK as a treatment for patients with stable COPD diagnosed with deficiency of lung qi and spleen qi.

### Design and setting

One-hundred patients with stable COPD are randomly assigned to two treatment groups (SHGBZK Chinese medicine treatment, *N* = 50; placebo treatment, *N* = 50). The two groups will receive basic treatment for COPD according to the 2017 GOLD Guidelines for Chronic Obstructive Pulmonary Disease. Both groups will receive a 24-week intervention and patient status is assessed at 24 weeks and then 28 weeks after treatment.

### Main outcome measures

The primary outcome is frequency of AECOPD. The secondary outcomes are duration of AECOPD, TCM symptom score, lung function, COPD Assessment Test (CAT) score, Modified Medical Research Council (MMRC) grade, BODE (Body mass index, airflow Obstruction, Dyspnea and Exercise capacity) score, and 6-min walking distance .

## Methods

### Study design

**Fig. 1:**
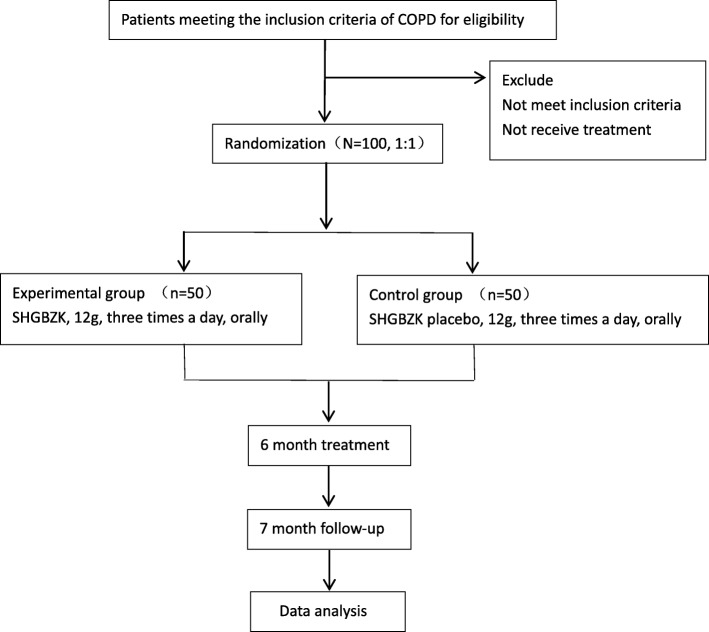
Flow chart of the study, a randomized, double-blind, placebo-controlled trial. A total of 100 patients will be recruited and randomly assigned to one of the two treatments group, with 50 in each group. One-hundred COPD patients will be randomly assigned to two treatment groups (SHGBZK treatment, *N* = 50; placebo treatment, N = 50). The two groups will receive basic treatment for COPD according to the 2017 GOLD Guidelines for Chronic Obstructive Pulmonary Disease. Patients will stick to the treatment they used to take as much as possible and will be given the trial treatment when AECOPD occurs during the study. Both groups will receive a 24-week intervention, and patient status will be assessed at 24 weeks and then 28 weeks after treatment. The outcome measures, including the frequency and duration of acute exacerbation, lung function, TCM symptom score, exercise capacity, and quality of life, will be assessed. Patients need to take medication as directed by the doctor. The use of glucocorticoids, antibiotics, mucolytic agents, and antitussive agents is prohibited during the study except if AECOPD occurs, and oral or external Chinese medicine preparations with the effect of tonifying the spleen and lung are prohibited during the trial period

We will conduct a randomized, double-blind, placebo-controlled trial. A flowchart of the study protocol is shown in Fig. 1.

### Ethics and recruitment

All patients will sign informed consent before inclusion. The study has been approved by the Ethics Research Committees of the China–Japanese Friendship Hospital with identifier 2018–57-K41–1. Any revisions of the study protocol will be submitted to the ethics committee.

COPD patients will be recruited from either the out-patient department or open recruitment. Recruitment began in November 2018 and will continue until a sample of 100 patients are enrolled.

### Inclusion criteria

The inclusion criteria are:

- The diagnostic criteria in the “Guidelines for the diagnosis and treatment of chronic obstructive pulmonary disease” (2013 revision) [13]
- Patients with stable symptoms such as cough, sputum production, or breathlessness for 4 weeks with no AECOPD
- The risk assessment of AECOPD is high (in the past year, two or more AECOPD occurrences or hospitalized at least once due to AECOPD)
- TCM syndrome pattern of lung qi and spleen qi deficiency as per the Guidelines for TCM Diagnosis and Treatment of Chronic Obstructive Pulmonary Disease (2011 edition) [14]
- Aged between 40 and 80 years
- With informed signed consent and voluntary participation in the study

### Exclusion criteria

Exclusion criteria include:

- Confirmed diagnosis of pneumonia and/or moderate to severe AECOPD in the past 4 weeks
- Accepted pneumonectomy in the past or lung volume reduction surgery in the 12 months before screening
- Accepted long-term oxygen therapy (time > 15 h/day) or mechanical aerator
- Patients with a history of asthma, active tuberculosis, lung cancer, bronchiectasis, pulmonary embolism, pulmonary heart disease, interstitial lung disease, or other active diseases
- Patients with lower extremity activity limitation and unable to complete the 6-minute walk test
- Patients diagnosed with serious hypertension, diabetes, tumors, or primary heart, liver, kidney, or blood system disease
- Scr exceeds the upper limit of the reference value by 1.5 times, or AST/ALT ratio ≥ 2 times the upper limit of the reference value
- Patients with congenital or acquired immunodeficiency disease
- Patients who are known or suspected of a history of alcohol or drug abuse
- Patients with confusion, dementia, or any kind of mental illness
- Pregnant or breast-feeding women
- Allergic to the used medicine
- Frequent use of oral glucocorticosteroids
- Patients enrolled in other clinical trials during the previous 3 months
- Anyone researchers believe should not participate in the clinical trial

### Withdrawal, dropout, and discontinuation

Participants are free to withdraw at any time during the trial. Participants who wish to withdraw will be offered the option to cease trial medication but continue attending scheduled visits for outcome measurement. Participants who withdraw will be followed to investigate the reason for withdrawal. Participants may be advised to discontinue the treatment if there is a product-related adverse event of a serious nature or if the participant was not compliant with the study requirements. Discontinuers will not be replaced by new participants. Intention-to-treat analysis will be performed on missing data from discontinuers with the last observation carried forward method.

### Sample size

A total of 100 patients will be enrolled in this study with 50 in each group. The frequency of AECOPD is the primary outcome. According to previous studies [15, 16], the exacerbation frequency increased 1.17 times each year when receiving conventional medicine, 0.97 times each year when receiving TCM, and 0.68 times each year when receiving both conventional medicine and TCM. Assume that promotional value is achieved only when the exacerbation frequency decreases at least once for each patient every 6 months. The standard deviation (SD) value is 1.25 times per year, the two-sided α is 0. 05, and β is 0. 10. Based on the formula:

$$ \left(\frac{2{\left({\mu}_{\alpha }+{\mu}_{\beta}\right)}^2{\sigma}^2}{\delta^2}\right) $$

of the comparison between the means of the two samples, the sample size in each group is 40. Considering a 20% dropout rate over the course of the study, 50 patients will be enrolled in each group and the total sample size will be 100.

### Randomization and masking

#### Randomization

The block randomization method will be used. The appropriate segment length will be selected and SAS9.4 (SAS Institute Inc., Cary, NC, USA) used to generate a randomization sequence for 100 subjects (test group, control group) according to a 1:1 ratio and list the treatment allocation corresponding to serial numbers 001–100 (that is, a random coding table). The placebo is made of SHGBZK Chinese medicine (5%) and dextrin (95%) to ensure it mimics the appearance, smell, and taste of SHGBZK. Both researchers and participants will not know the assignment. The randomization sequence table will be kept in a file. The method, process, group setting, and grouping result of the randomization sequence will be recorded so it can be checked when necessary. Information on intervention assignments will be kept in the third consulting center of biomedical statistics.

#### Blinding

Design: In this study, two stage blinding is used. The first stage blinding is represented by groups A and B. The second stage blinding is represented by the corresponding test drug and placebo.

Blinding management and preservation: Blinding is carried out by the statistical unit. The clinical trial unit and the statistical analysis unit are deposited in accordance with the relevant regulations after the blinding is sealed. The process of drug coding will be written by the blinder and saved.

Emergency unblinding: If an adverse event occurs during the study, the main investigator can decide whether to unblind according to the subject. The investigator needs to record the time, location, and cause of the unblinding in the medical record and Case Report Form (CRF) (the group information after unblinding should not be recorded in the CRF).

### Intervention measures

The two groups will receive basic treatment for COPD according to the 2017 GOLD Guidelines for Chronic Obstructive Pulmonary Disease. Patients will stick to the treatment they used to take as much as possible and will be given the trial treatment when AECOPD occurs during the study. Patients in the experimental group will take SHGBZK, while the control group will take SHGBZK placebo. The TCM granules are compound preparations of Chinese herbs; its main components are shown in Table 1 . Each bag of SHGBZK granules (batch number 180606) contains 3 g. The components of the TCM granules are produced and packed by An Hui Ji Ren Pharmaceutical Co. Ltd according to Good Manufacturing Practice (approval number AH20160363), Anhui, PR China. The test results of drug quality were consistent with the required quality standards. Each type of granule will be given orally, four bags each time, three times a day for 24 weeks.

**Table 1:** Main components of the traditional Chinese medicine treatment

Chinese name	Latin name	Amount (g)
Chinese herbal formula San-Huang Gu-Ben Zhi-Ke		
Huang Qi	*Astragalus propinquus*	15
Huang Jing	*Polygonatum sibiricum*	12
Chen Pi	*Pericarpium citri reticulatae*	10
Bai Bu	*Stemona japonica*	10
Wu Wei Zi	*Schisandra chinensis*	8
Chi Shao	*Paeonia lactiflora* Pall	10
Huang Qin	*Scutellaria baicalensis* Georgi	8

Patients need to take the medication as directed by the doctor. The use of glucocorticoids, antibiotics, mucolytic agents, and antitussive agents is prohibited during the study except if AECOPD occurs, and oral or external Chinese medicine preparations with the effect of tonifying the spleen and lung are prohibited during the trial period. Patients will be given a daily diary to record their trial medication compliance as well as use of any other therapies and occurrence of adverse events. Patients will be asked to return their medication bags monthly during the treatment period to enable the counting of left-over capsules as well as part of participant adherence monitoring.

### Outcome measures

#### Primary outcome measure

The frequency of AECOPD is the primary outcome measure. AECOPD is characterized by increased respiratory symptoms beyond daily routine variation and requires a change in regular medication. Its reduction is a major goal of COPD management and an important indicator for evaluating the treatments. AECOPD is considered if at least two major symptoms or one major symptom plus more than one minor symptoms occur: major symptoms are increased difficulty in breathing, increased sputum volume, purulent sputum; minor symptoms are upper respiratory tract infection, unexplained fever, and wheezing. If the interval between two onsets of acute exacerbation is less than 1 week and the acute exacerbation lasts for at least 2 days, it is counted as one acute exacerbation event. The frequency and duration of AECOPD occurrences during the 24-week treatment period and 7-month follow-up will be counted and the total number and average frequency and duration determined.

#### Secondary outcome measures

##### AECOPD

Time of first AECOPD occurence, the interval between two onsets of acute exacerbation, the duration of AECOPD, and the severity of AECOPD after the treatment.

##### TCM symptom score

The TCM symptom score for patients with stable COPD diagnosed with deficiency of lung qi and spleen qi will be adopted. The TCM symptom score scale is scored from 0 (normal) to 22 (severe). The TCM symptom score is shown in Table 2.

**Table 2:** TCM symptom score

Main symptom (score)	Normal (0)	Light (2)	Medium (4)	Severe (6)
Cough	No	Intermittent cough during the day	Cough during day and night without affecting work and sleep	Cough frequently during day and night which affects work and sleep
Sputum	No	A small amount of sputum	Sputum and wheezy phlegm	A large amount of sputum and loud wheezy phlegm
Shortness of breath	No	Shortness of breath after work	Fatigue and shortness of breath	Short of breath when quiet
Secondary symptoms	Normal (0)	Light (1)	Medium (2)	Severe (3)
Spontaneous perspiration	No	Sweat while eating	Intermittent sweat	Sweat soaks clothes, sweat more after work
Loss of appetite	No	Loss of appetite, but eat as usual	Eat less but not under one-third of usual	Eat less than one-third of usual
Weak	No	Can do light physical work	Can’t do physical work	General fatigue, intend to stay in bed
Abdominal distention and loose stool	No	Light abdominal distention and the stool is not forming	Abdominal distention is obvious and loose stool	Abdominal distention is obvious and water-like stool

##### Lung function

Forced vital capacity (FVC), forced expiratory volume in 1 s (FEV1), forced expiratory volume in 1 s (FEV1% pred), FEV1/FVC, maximum expiratory mid-flow (MMEF), and peak expiratory flow (FEF) will be tested. A positive change from baseline in these will indicate an improvement in lung function.

The Modified Medical Research Council (MMRC) scale by the American Thoracic Society [17] will be assessed to evaluate the level of dyspnea. The MMRC scale is a simple grading system that scores from 0 (less severe) to 4 (severe).

##### Quality of life

The COPD Assessment Test (CAT) will be adopted. The CAT is a self-complete questionnaire with eight items, each formatted as a 6-point semantic differential scale ranging from 0 to 5. CAT scores range from 0 to 40. Higher scores denote a more severe impact on a patient’s quality of life. The patients will be invited to complete the questionnaires through face-to-face survey. The patients can answer each question and check the most appropriate opinion (a specific score) with regards to their standards, hopes, pleasures, and concerns. Meanwhile, an investigator in each center will be assigned in the office to help the patients and to check through each completed questionnaire to ensure that the patients have answered all the questions.

##### The 6-min walking distance

The 6-min walking distance is used to evaluate the distance a person can walk on a flat surface in 6 min to assess their exercise capacity. The BODE index will be used; BODE stands for Body mass index, airflow Obstruction, Dyspnea and Exercise capacity. BODE scores range from 0 to 40 and will be further quartilized as follows: quartile 1 (a score of 0 to 2 points), quartile 2 (a score of 3 to 4 points), quartile 3 (a score of 5 to 6 points), and quartile 4 (a score of 7 to 10 points). The higher the level, the worse the patient’s condition.

##### Concomitant medication status

Drug therapies used to treat COPD during the study will be recorded.

##### Mortality

All-cause mortality and COPD mortality will be calculated for the subjects during the study.

##### Safety

Routine blood, urine, and stool tests, liver and kidney function tests, and an electrocardiogram will be performed. Adverse events will be recorded at any time during the treatment period and follow-up period.

Adverse events will be recorded and graded in detail throughout the study, such as possible side effects (no side effects of the herbs have been reported so far). If a severe adverse event occurs, participants will be provided with every necessary treatment, and the event must be reported to the leader of the trial, ethics committees, sponsors, and China Food and Drug Administration (CFDA) within 24 h.

### Screening and run-in, baseline, treatment periods, and endpoint

**Fig. 2:**
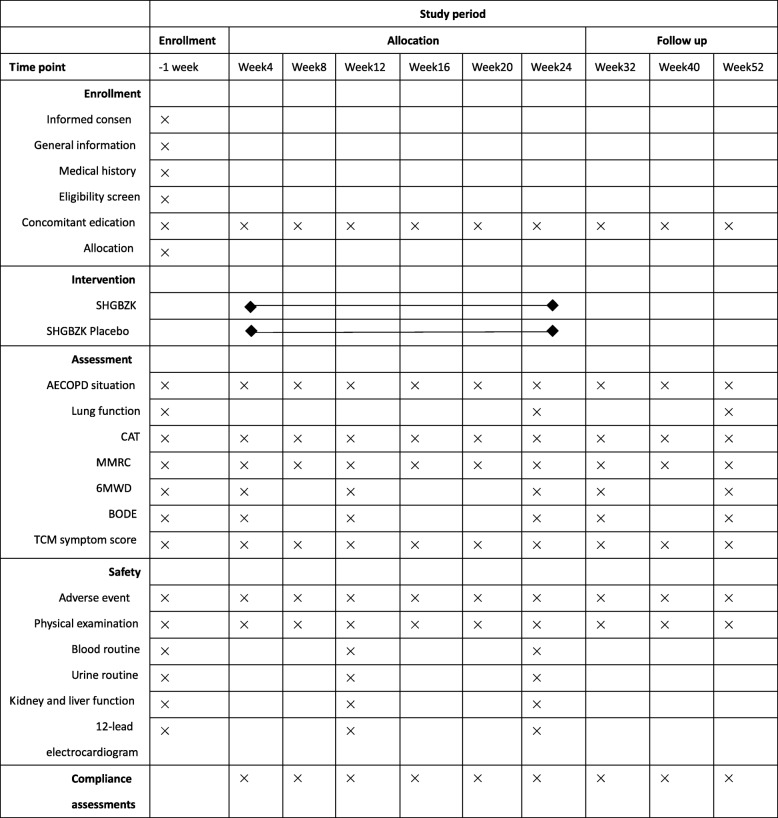
Schedule of enrollment, intervention, and assessments. Adverse events, physical examination, AECOPD situation and MMRC, CAT, and TCM symptom scores (TCM symptoms of patients with stable COPD diagnosed with deficiency of lung qi and spleen qi) will be recorded at baseline (week 0) and every 4 weeks during the study period. The 6-min walking distance and BODE will be recorded at weeks 0, 4, 12, 24, 32, and 52. Lung function will be determined at weeks 0, 24, and 52. Safety will be measured at weeks 0, 12, and 24, except for adverse events and physical examination

Adverse events, physical examination, AECOPD situation and MMRC, CAT, and TCM symptom scores will be recorded at baseline (week 0) and every 4 weeks during the study period. The 6-min walking distance and BODE will be recorded at weeks 0, 4, 12, 24, 32, and 52. Lung function will be observed at weeks 0, 24, and 52. Safety will be measured at weeks 0, 12, and 24, not including adverse events and the physical examination. The schedule of assessments and interventions is depicted in Fig. 2.

### Statistical analysis

All data will be analyzed by an independent statistician using SAS 9.4. For all analyses, *P* < 0.05 is considered statistically significant. Measurement data will be presented as number of cases, mean, standard deviation, minimum, median, maximum, upper quartile (Ql), lower quartile (Q3), and 95% confidence interval (95% Cl) data. Paired-sample *t*-test or signed rank sum test will be used to compare the difference between the two groups or pretreatment and posttreatment within one group. Analysis of covariance will be used to compare the differences of center effector other confounding factors.

## Discussion

Syndrome differentiation is the basic principle of Chinese medicine to recognize and treat diseases. It is held that the efficacy of TCM is most obviously observed in alleviating or improving the characteristic symptoms of patients diagnosed with the corresponding TCM syndrome. Professor Chao Enxiang, a great doctor of Chinese medicine, attaches great importance to the position and role of the body’s vital energy in the pathogenesis of COPD. He emphasizes that “positive Qi exists in the body, thus evil Qi cannot invade” [18]. He believes that deficiency of lung and spleen and obstruction of phlegm and turbidity are the main pathogenesis characteristics of stable COPD. SHGBZK was developed for tonifying the lung, strengthening the spleen, resolving phlegm, and relieving cough.

According to previous clinical studies [11], the Chinese medicine SHGBZK has a good clinical effect (100%) with regards to improving the effective rate of cough and cough in patients, improving wheezing efficiency by 85.71%. The total effective rate of SHGBZK is 96.67%, and no side effects have been found so far. In our study, we will conduct a randomized, double-blind, placebo-controlled trial to evaluate the safety and efficacy of the Chinese herbal formula SHGBZK for stable COPD patients diagnosed with deficiency of lung qi and spleen qi. This study may establish a new treatment method for stable COPD patients, differentiating it from other drugs in clinical use for similar clinical indications.

In our study, the frequency of acute AECOPD has been chosen as the primary outcome. We also employ validated and objective tools, such as the ACT score and FEV1, as outcome measurements. These measurements improve the reliability and generalizability of the results. Measures will be taken to strengthen quality control. To avoid bias from researchers during this study, an investigator separate from all of the clinical researchers will be hired to preserve and record the randomization information. Therefore, the clinical researchers will not have any input to enrollment or randomization. Also, outcome assessments will be made by an independent clinical statistician blinded to group allocation and not involved in providing intervention or management. We have built a TCM symptom score table to evaluate the TCM symptoms of patients with stable COPD diagnosed with deficiency of lung qi and spleen qi. However, considering the difficulty of recruitment because of the strict inclusion criteria, we have adopted the minimum sample size. Thus, the sample size is a little too small to observe changes in lung function and then show an effect of SHGBZK treatment.

The study has been developed according to the Consolidated Standards of Reporting Trials (CONSORT) [17] statement.

## Data Availability

Not applicable.
